# Association between pan-immune inflammatory value and all-cause mortality in critically ill patients with ischemic stroke: a retrospective analysis of the MIMIC-IV database (2008–2022)

**DOI:** 10.3389/fneur.2025.1644817

**Published:** 2025-08-18

**Authors:** Anni Chen, Jiahui Wang, Qiaoying Huang, Zhizhou Hu, Donglan Jiang, Yunnan Hu, Liying Pan, Jianhui Chen, Xiaohong Hu

**Affiliations:** Department of Neurology, Longyan First Affiliated Hospital of Fujian Medical University, Longyan, China

**Keywords:** cerebrovascular disease, all-cause mortality, MIMIC-IV, inflammatory biomarker, stroke

## Abstract

**Background:**

Systemic inflammation and immune responses are key contributors to the onset and progression of ischemic stroke, influencing both tissue damage and repair. This study investigates the association between the pan-immune inflammatory value (PIV)—a composite biomarker derived from routine blood tests—and all-cause mortality (ACM) in critically ill ischemic stroke patients.

**Methods:**

We extracted data from the MIMIC-IV (v3.0) database, identifying ischemic stroke patients using ICD-9/10 codes. PIV was calculated using the formula: (monocytes × neutrophils × platelets) ÷ lymphocytes. ACM was assessed during hospitalization and at 30-, 90-, and 365-days post-admission. Multivariable Cox proportional hazards models and restricted cubic spline (RCS) analyses were used to assess the relationship between PIV and mortality. Kaplan–Meier curves, time-dependent ROC curves, and decision curve analysis (DCA) evaluated survival differences and predictive performance. Subgroup and interaction analyses were conducted using likelihood ratio tests.

**Results:**

A total of 1,365 critically ill ischemic stroke patients were included, with 50.48% male. Elevated PIV was significantly associated with higher mortality during hospitalization (HR: 1.98), and at 30-day (HR: 2.56), 90-day (HR: 1.97), and 365-day (HR: 1.76) follow-ups (all *p* < 0.01). RCS analysis revealed a J-shaped relationship between PIV and ACM. Subgroup analyses showed consistent results without significant interaction effects.

**Conclusion:**

PIV is an independent predictor of short- and long-term mortality in critically ill ischemic stroke patients. These findings suggest PIV could serve as a practical and cost-effective biomarker for risk stratification and prognosis in clinical settings.

## Highlights

- Systemic inflammation and immune responses are key contributors to stroke onset, progression, and recovery, highlighting the need for reliable inflammatory biomarkers.
- This study examined the pan-immune inflammatory index (PIV) as a predictor of all-cause mortality (ACM) in critically ill ischemic stroke patients using data from the MIMIC-IV database (*N* = 1,365).
- Higher PIV levels were significantly associated with increased mortality at all measured time points: during hospitalization, and at 30, 90, and 365 days post-admission (all *p*-values ≤0.01).
- A J-shaped relationship between PIV and mortality risk was observed using restricted cubic spline analysis, and the findings remained robust across various subgroups.
- These results support PIV as an independent prognostic biomarker for mortality risk in ischemic stroke, potentially aiding clinical decision-making in critical care settings.

## Introduction

According to the 2021 Global Burden of Disease (GBD) data, stroke remains the second leading cause of death and the third leading cause of disability from non-communicable diseases (NCDs) worldwide (1). Between 1990 and 2021, the number of people suffering from stroke, dying from stroke, or becoming disabled after a stroke significantly increased, as indicated by the 70% increase in the incidence of stroke and the 44% increase in the mortality rate (2). Relevant studies predict that by 2050, the population aged 65 and over will exceed 150 million, and the burden of stroke will grow in tandem with the aging population (3). To alleviate the disease burden caused by stroke, it is crucial to identify prognostic indicators that can predict adverse outcomes for stroke patients.

Inflammation is a significant contributing factor for atherosclerosis, thrombosis, and small vessel diseases in the brain. Subsequently, these key mechanisms may lead to large artery atherosclerosis, cardioembolic events, lacunar and cryptogenic ischemic strokes (4). After a stroke, local brain tissue damage triggers an inflammatory cascade by producing immune-active molecules (damage-associated molecular patterns, DAMPs) that exacerbate local tissue injury and suppress the body’s immune response, subsequently inducing a series of adverse reactions and increasing the risk of patient mortality (5). Moreover, secondary inflammatory immune responses can persist for months or even years, spreading throughout the body and facilitating stroke-related sequelae (5). During the onset and progression of ischemic stroke, neutrophils, monocytes, platelets, and lymphocytes collectively participate in the complex inflammatory immune response. Neutrophils, important inflammatory cells, are recruited to the injured site early in stroke, rapidly infiltrating damaged tissues and releasing pro-inflammatory cytokines, forming extracellular traps (NETs) and intensifying inflammation and thrombosis (6–9). Monocytes, after being induced to enter the damaged brain tissue by chemokines (CCL2/CCR2), play a critical role in regulating inflammatory responses and tissue repair (10–12). After a stroke, activated platelets form aggregates through interactions with monocytes, neutrophils, and lymphocytes, leading to excessive coagulation function, increasing the risk of thrombosis, and exacerbating occlusion at the site of injury (13). Lymphocytes regulate inflammatory responses through adaptive immune reactions, where T cells play a dual role: they can exacerbate nerve damage and promote tissue repair via regulatory T cells (14–16). B cells influence the activity of other immune cells by producing antibodies and secreting cytokines, affecting inflammatory immune balance after stroke (17). The interactions among these cells determine the extent of neurological damage and the recovery process after stroke (18). Currently, numerous serological indicators, such as the neutrophil-to-lymphocyte ratio (NLR), monocyte-to-lymphocyte ratio (MLR), and platelet-to-lymphocyte ratio (PLR) are widely used in studies related to the clinical prognosis of stroke patients (19–22). PIV, a novel inflammatory burden index, was initially proposed by Fucà et al. (23) in a study on prognostic factors in first-line treatment of metastatic colorectal cancer. It demonstrates better predictive efficacy for the prognosis of rectal cancer patients than other inflammatory indicators (such as NLR, PLR), and also exhibits good predictive efficacy for other cancers besides rectal cancer (24). However, few studies have explored the correlation between PIV and the prognosis of stroke patients. The focus of this study was to investigate the predictive ability of PIV for mortality risk in critically ill patients with ischemic stroke using data from the extensive intensive care medical information market (MIMIC)-IV database.

## Materials and methods

### Study population

The data for this study was obtained from the MIMIC-IV database (v3.0), a comprehensive and publicly accessible database that contains de-identified health-related data from 94,458 ICU admissions at Beth Israel Deaconess Medical Center in Boston, Massachusetts, USA, from 2008 to 2022 (collected by the MIT Computational Physiology Laboratory). To ensure patient privacy, all personal information was anonymized using random codes. The database is publicly available through the PhysioNet online platform. To obtain it, the lead author, Anni Chen, completed the Collaborative Institutional Training Initiative (CITI) course and passed the “Conflict of Interest” and “Data or Sample Only Research” (ID: 13622063) exams. Following this, the research team was granted authorization to use and extract data from the database.

**Figure 1:**
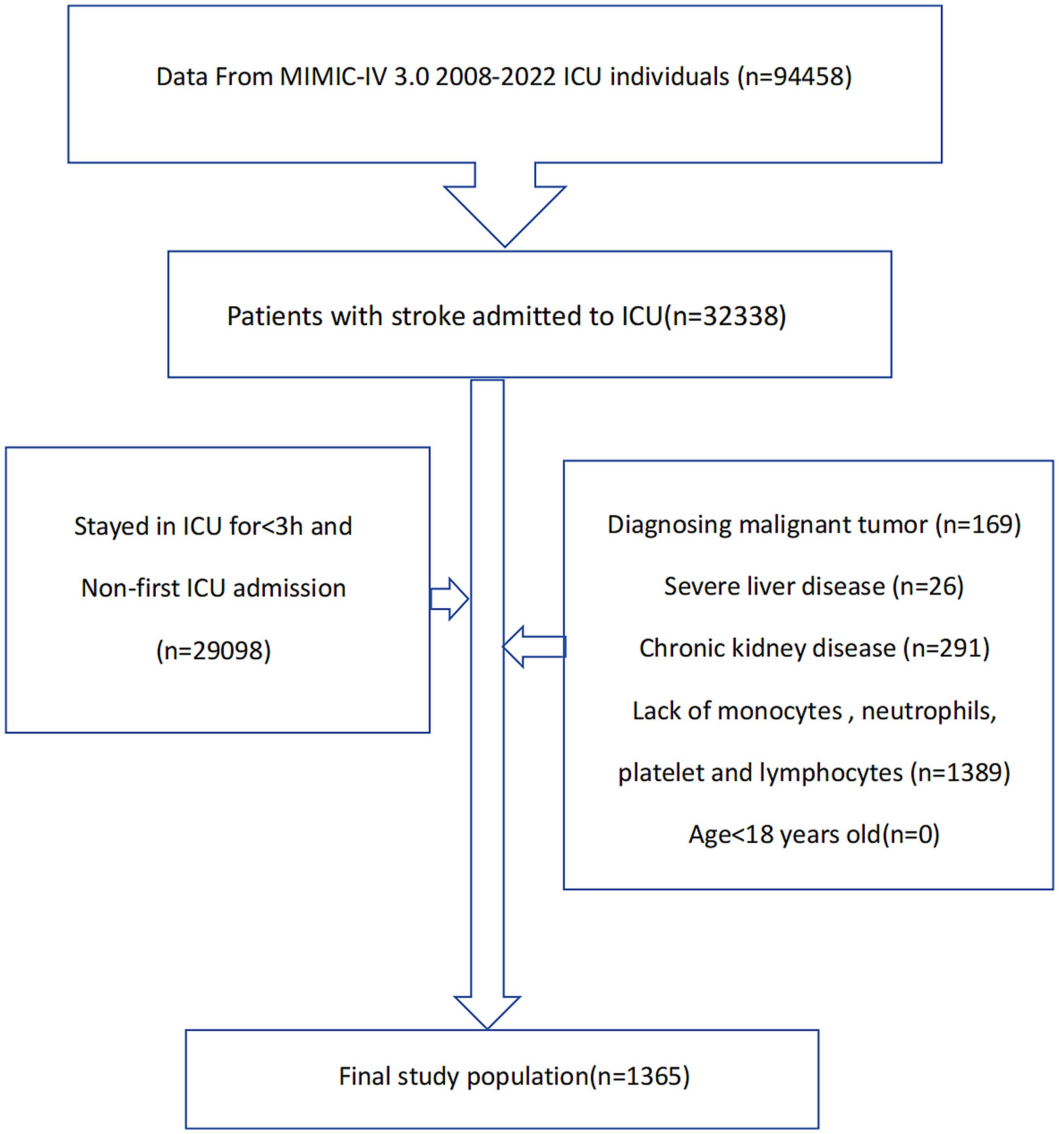
Flowchart of experimental design.

This study retrospectively included patients with acute ischemic stroke admitted to the ICU. Inclusion criteria were: (1) age over 18 years; (2) diagnosed as acute ischemic stroke according to ICD-9 and ICD-10 codes; (3) hospitalized in the ICU for at least 3 h; (4) first-time hospitalization or first admission to the ICU; (5) exclusion of patients with malignant tumors, chronic nephritis, and severe liver disease; (6) exclusion of patients with missing key data. Finally, this study included 1,365 patients (Figure 1).

### Data collection

To retrieve data from the database, PostgreSQL software (version 13.7.2) and Navicat Premium (version 16) were deployed. The process was simplified by applying Structured Query Language (SQL). This process targets data acquisition in five main areas: (1) Demographic statistics including age and gender, (2) Clinical severity indices such as the Glasgow Coma Scale (GCS), Sequential Organ Failure Assessment (SOFA) score, Simplified Acute Physiology Score II (SAPS-II), Acute Physiological and Chronic Health II (APSIII) score, Braden Assessment Scale (Braden), and Oxford Acute Disease Severity Score (OASIS). (3) Physiological indicators including body weight, mean arterial pressure (MBP), systolic blood pressure (SBP), diastolic blood pressure (DBP), temperature in degrees Celsius, heart rate, respiratory rate, and oxygen saturation (SPO_2_) measured by pulse oximeter. (4) Hematological and biochemical indicators including neutrophil count (NEU), lymphocyte count (LYM), platelet count (PLT), monocyte count (MONO), white blood cell count (WBC), prothrombin time (PT), international normalized ratio (INR), alanine transaminase (ALT), aspartate transaminase (AST), and alkaline phosphatase (ALP). (5) Existing comorbidities, such as hypertension, diabetes, hyperlipidemia, heart failure (HF), chronic obstructive pulmonary disease (COPD), Charlson comorbidity index (CCI), as well as therapeutic interventions including antiplatelet drugs, anticoagulants, intravenous thrombolysis, and vascular interventional therapy. The observation spans for each case extended from admission to the time of death event. The PIV calculation formula was: (monocytes × neutrophils × platelets) ÷ lymphocytes. This analysis relied on laboratory values and scores that indicated disease severity, which are typically collected within 24 h of a patient’s admission to the ICU. To eliminate the impact of missing data, variables with a missing data rate exceeding 20% were excluded to reduce bias introduced by other variables (25).

### Clinical outcomes

The clinical endpoints of this study were hospitalization, 30-day, 90-day, and 365-day ACM of acute ischemic stroke patients admitted to the ICU.

### Statistical analysis

This study stratified participants into quintiles based on their PIV values (Q1–Q5). Quantitative variables were reported as mean ± standard deviation (SD) or median and interquartile range, depending on the data distribution. Qualitative variables were presented as counts and proportions. For continuous variables that followed a normal distribution, *t*-tests or ANOVA were used for analysis, while Mann–Whitney *U* tests or Kruskal–Wallis tests were applied to variables that deviated from normality. The Kaplan–Meier (KM) survival method was applied to determine the incidence of endpoints within groups defined by PIV levels, and log-rank test was used to assess statistical differences. The Cox proportional hazards model was used to quantify the association between PIV and the study endpoint, generating risk ratios (HR) and 95% confidence intervals (CI). Three models were combined to adjust for confounding factors: Model 1 (adjusted for age and sex), Model 2 (adjusted for Model 1 and hypertension, diabetes, hyperlipidemia, atrial fibrillation, chronic obstructive pulmonary disease (COPD), acute heart failure (HF), prothrombin time (PT), international normalized ratio (INR), alanine transaminase (ALT), aspartate transaminase (AST) and alkaline phosphatase (ALP)), and Model 3 [adjusted for Model 2 and the Glasgow Coma Scale (GCS), antithrombotic drugs, anticoagulants, intravenous thrombolysis, vascular intervention, and Charlson comorbidity index (CCI)]. In addition, the efficacy of the prediction model was described by drawing time-dependent ROC curve and DCA clinical decision curve. To further explore the relationship between PIV and mortality risk in critically ill patients with ischemic stroke, restricted cubic splines (RCS) were used to construct a Cox proportional hazards model to precisely identify any nonlinear associations. Stratified and interaction analyses examined the effects of sex, age (under 65 or 65 and older), presence of hypertension, diabetes, heart failure (HF), anticoagulation, intravenous thrombolysis, and GCS score ≥8. Likelihood ratio tests were used to detect interactions. The HRs in subgroups were the same as those in Model 3. All analyses were conducted with a significance threshold of *p* < 0.05 (two-tailed), using R software (version 4.2.2) and SPSS 22. 0.

## Results

The analysis included 1,365 patients, with males accounting for 50.48%. Multivariate Cox regression analysis revealed that elevated PIV was significantly associated with critical ischemic stroke patients in hospital [risk ratio (HR): 1.98; 95% confidence interval CI: (1.19–3.29); *p* = 0.01], at 30 days [risk ratio (HR): 2.56; 95% confidence interval CI: (1.65–3.97); *p* < 0.0001], at 90 days [risk ratio (HR): 1.97; 95% confidence interval CI: (1.35, 2.89); *p* < 0.001], and at 365 days after initial hospitalization [risk ratio (HR): 1.76; 95% confidence interval CI: (1.25–2.48); *p* = 0.001]. The RCS analysis results showed that PIV exhibited a “J-shaped curve relationship” with ACM in ischemic stroke patients. Interaction tests found that this relationship was not significantly affected by differences among subgroups.

### Baseline characteristics

Table 1 lists the baseline demographics and clinical attributes stratified by PIV quintiles. Participants were divided into five quintiles (Q1: 3.224–302.372; Q2: 302.372–605.696; Q3: 605.696–1052.635; Q4: 1052.635–2017.179; Q5: 2017.179–44699.253). We observed that individuals in the highest quintile of PIV exhibited higher vital signs such as body temperature, heart rate, and respiratory rate (RP), while having lower mean arterial pressure (MBP), systolic blood pressure (SBP), and diastolic blood pressure (DBP). Additionally, they had higher Sequential Organ Failure Assessment (SOFA) scores, Simplified Acute Physiology (SAPSII) scores, Acute Physiological and Chronic Health II (APSIII) scores, and Oxford Acute Disease Severity Index (OASIS) scores, as well as lower Braden Assessment Scale (Braden) scores. These patients also showed higher neutrophil count (NEU), platelet count (PLT), monocyte count (MONO), white blood cell count (WBC), prothrombin time (PT), international normalized ratio (INR), alanine transaminase (ALT), aspartate transaminase (AST), and alkaline phosphatase (ALP) levels, and lower lymphocyte count (LYM). Compared with the lower quintile group, there was a higher proportion of vascular interventional therapy, accompanied by higher mortality in ICU, hospitalization and post-hospitalization at 30 days, 90 days, and 365 days. The group with high PIV had a large proportion of patients receiving endovascular interventional treatment, and the mortality rate was also high, which may be due to either of two reasons. First, the higher the PIV, the greater the degree of inflammatory immune imbalance in the body, and consequently, the more severe the disease, increasing the likelihood of requiring endovascular intervention. Alternatively, a higher PIV may indicate a more severe degree of inflammatory response and immune suppression. Neutrophils release metalloproteinases (MMP-9), which degrade the extracellular matrix, increasing the risk of hemorrhagic transformation after endovascular intervention. Platelet hyperreactivity and monocyte involvement in unstable plaque formation collectively lead to revascularization without recanalization. Additionally, reduced lymphocytes result in immune suppression, increasing the risk of infection. All these factors contribute to the high mortality rate in individuals with elevated PIV, even after receiving endovascular intervention.

**Table 1:** The baseline demographics and clinical attributes stratified by PIV quintiles.

Variable	Total (*n* = 1,365)	Q1 (*n* = 273)	Q2 (*n* = 273)	Q3 (*n* = 273)	Q4 (*n* = 273)	Q5 (*n* = 273)	*p*-value
Sex							0.24
Female	676 (49.52)	141 (51.65)	139 (50.92)	134 (49.08)	143 (52.38)	119 (43.59)	
Male	689 (50.48)	132 (48.35)	134 (49.08)	139 (50.92)	130 (47.62)	154 (56.41)	
Age							0.15
>65 years	805 (58.97)	163 (59.71)	176 (64.47)	147 (53.85)	157 (57.51)	162 (59.34)	
≤65 years	560 (41.03)	110 (40.29)	97 (35.53)	126 (46.15)	116 (42.49)	111 (40.66)	
Weight	80.65 ± 23.17	78.51 ± 21.88	79.72 ± 23.53	83.03 ± 23.19	81.18 ± 24.13	80.78 ± 22.98	0.21
Heart rate	80.78 ± 16.25	77.26 ± 15.95	77.48 ± 14.86	79.61 ± 15.02	83.56 ± 16.44	86.01 ± 17.10	**<0.0001**
SBP	130.48 ± 19.08	130.62 ± 20.07	133.35 ± 18.80	131.54 ± 19.43	130.19 ± 18.32	126.70 ± 18.23	**<0.01**
DBP	71.69 ± 13.06	72.51 ± 13.33	72.88 ± 12.88	72.71 ± 12.49	71.86 ± 13.08	68.48 ± 13.06	**<0.001**
MBP	88.37 ± 13.16	89.01 ± 13.71	89.95 ± 12.87	89.41 ± 12.91	88.39 ± 12.99	85.08 ± 12.85	**<0.001**
RP	18.82 (16.96, 21.19)	18.21 (16.74, 20.33)	18.12 (16.56, 20.17)	18.38 (16.79, 20.49)	19.13 (17.29, 21.36)	19.97 (17.84, 22.83)	**<0.0001**
T	36.88 (36.70, 37.13)	36.82 (36.68, 37.00)	36.86 (36.69, 37.01)	36.90 (36.72, 37.14)	36.92 (36.72, 37.17)	36.99 (36.74, 37.34)	**<0.0001**
SPO_2_	97.00 (95.67, 98.29)	97.28 (95.88, 98.43)	96.83 (95.70, 98.24)	96.80 (95.52, 98.04)	96.85 (95.48, 98.15)	97.39 (95.83, 98.67)	0.08
Hypertension							0.16
No	620 (45.42)	135 (49.45)	112 (41.18)	117 (42.70)	121 (44.32)	135 (49.45)	
Yes	745 (54.58)	138 (50.55)	160 (58.82)	157 (57.30)	152 (55.68)	138 (50.55)	
Fibration							0.34
No	1,221 (89.45)	242 (88.64)	236 (86.45)	250 (91.58)	246 (90.11)	247 (90.48)	
Yes	144 (10.55)	31 (11.36)	37 (13.55)	23 (8.42)	27 (9.89)	26 (9.52)	
Diabetes							0.07
No	1,175 (86.08)	232 (84.98)	222 (81.32)	239 (87.55)	238 (87.18)	244 (89.38)	
Yes	190 (13.92)	41 (15.02)	51 (18.68)	34 (12.45)	35 (12.82)	29 (10.62)	
Hyperlipemia							0.30
No	1,260 (92.31)	256 (93.77)	247 (90.48)	248 (90.84)	251 (91.94)	258 (94.51)	
Yes	105 (7.69)	17 (6.23)	26 (9.52)	25 (9.16)	22 (8.06)	15 (5.49)	
HF							0.07
No	1,153 (84.47)	238 (87.18)	239 (87.55)	226 (82.78)	232 (84.98)	218 (79.85)	
Yes	212 (15.53)	35 (12.82)	34 (12.45)	47 (17.22)	41 (15.02)	55 (20.15)	
COPD							**<0.001**
No	1,079 (79.05)	230 (84.25)	228 (83.52)	223 (81.68)	199 (72.89)	199 (72.89)	
Yes	286 (20.95)	43 (15.75)	45 (16.48)	50 (18.32)	74 (27.11)	74 (27.11)	
PLT	224.00 (176.00, 281.00)	182.00 (144.00, 232.00)	211.00 (171.00, 254.00)	231.00 (187.00, 280.00)	231.00 (183.00, 289.00)	282.00 (226.00, 338.00)	**<0.0001**
LYM	1.39 (0.95, 1.95)	1.77 (1.29, 2.42)	1.54 (1.14, 2.19)	1.40 (1.05, 1.88)	1.24 (0.90, 1.67)	1.01 (0.72, 1.45)	**<0.0001**
MONO	0.63 (0.44, 0.88)	0.40 (0.29, 0.53)	0.53 (0.38, 0.71)	0.61 (0.48, 0.78)	0.76 (0.61, 1.03)	1.06 (0.78, 1.39)	**<0.0001**
NEU	8.12 (5.64, 11.63)	4.42 (3.29, 5.55)	6.32 (5.21, 7.85)	7.84 (6.57, 9.64)	10.04 (8.43, 12.71)	13.92 (10.92, 18.08)	**<0.0001**
WBC	10.90 (8.10, 14.50)	7.60 (6.00, 10.60)	9.20 (7.60, 11.50)	10.38 (8.60, 12.60)	12.40 (9.90, 15.30)	15.80 (12.20, 20.20)	**<0.0001**
INR	1.20 (1.10, 1.34)	1.20 (1.10, 1.36)	1.20 (1.10, 1.30)	1.20 (1.10, 1.30)	1.20 (1.10, 1.34)	1.20 (1.10, 1.40)	**<0.0001**
PT	13.10 (12.00, 14.80)	13.20 (11.90, 14.90)	12.70 (11.90, 13.76)	12.90 (11.90, 14.30)	13.10 (11.90, 14.90)	13.80 (12.50, 15.60)	**<0.0001**
ALT	23.00 (15.00, 40.00)	20.60 (15.00, 32.00)	21.00 (15.00, 30.20)	21.00 (14.00, 34.00)	26.00 (16.00, 47.00)	29.00 (17.00, 60.00)	**<0.0001**
ALP	78.80 (63.00, 96.80)	78.00 (61.00, 93.00)	77.00 (64.00, 92.00)	75.00 (62.00, 92.00)	82.00 (64.60, 102.00)	85.00 (66.00, 105.40)	**<0.001**
AST	31.00 (21.00, 58.00)	29.00 (20.00, 47.00)	28.00 (20.00, 42.00)	29.00 (19.00, 45.00)	34.00 (23.00, 69.00)	44.00 (25.00, 99.00)	**<0.0001**
GCS							0.26
<8	115 (8.42)	20 (7.33)	16 (5.86)	24 (8.79)	25 (9.16)	30 (10.99)	
≥8	1,250 (91.58)	253 (92.67)	257 (94.14)	249 (91.21)	248 (90.84)	243 (89.01)	
CCI	6.00 (4.00, 7.00)	5.00 (4.00, 7.00)	6.00 (4.00, 7.00)	5.00 (4.00, 7.00)	6.00 (4.00, 7.00)	6.00 (4.00, 7.00)	0.61
SOFA	3.00 (1.00, 5.00)	2.00 (1.00, 5.00)	2.00 (1.00, 4.00)	3.00 (1.00, 5.00)	3.00 (2.00, 5.00)	4.00 (2.00, 6.00)	**<0.0001**
OASIS	31.48 ± 8.50	30.20 ± 8.85	29.40 ± 7.86	30.99 ± 8.10	32.26 ± 8.66	34.55 ± 8.11	**<0.0001**
SAPSII	31.00 (24.00, 40.00)	29.00 (23.00, 37.00)	29.00 (21.00, 37.00)	29.00 (23.00, 37.00)	32.00 (25.00, 40.00)	35.00 (28.00, 45.00)	**<0.0001**
Braden	14.00 (12.00, 16.00)	15.00 (13.00, 17.00)	15.00 (13.00, 17.00)	14.00 (12.00, 16.00)	14.00 (12.00, 15.00)	12.00 (11.00, 14.00)	**<0.0001**
APSIII	35.00 (26.00, 48.00)	33.00 (25.00, 43.00)	31.00 (25.00, 43.00)	34.00 (26.00, 44.00)	36.00 (28.00, 50.00)	43.00 (32.00, 56.00)	**<0.0001**
Antiplate							0.06
No	301 (22.05)	51 (18.68)	57 (20.88)	58 (21.25)	57 (20.88)	78 (28.57)	
Yes	1,064 (77.95)	222 (81.32)	216 (79.12)	215 (78.75)	216 (79.12)	195 (71.43)	
Anticoagulation							0.08
No	468 (34.29)	87 (31.87)	77 (28.21)	100 (36.63)	105 (38.46)	99 (36.26)	
Yes	897 (65.71)	186 (68.13)	196 (71.79)	173 (63.37)	168 (61.54)	174 (63.74)	
Surgery							**<0.0001**
No	276 (20.22)	71 (26.01)	69 (25.27)	63 (23.08)	40 (14.65)	33 (12.09)	
Yes	1,089 (79.78)	202 (73.99)	204 (74.73)	210 (76.92)	233 (85.35)	240 (87.91)	
Thrombolysis							0.20
No	1,287 (94.29)	258 (94.51)	250 (91.91)	265 (96.72)	258 (94.51)	256 (93.77)	
Yes	78 (5.71)	15 (5.49)	22 (8.09)	9 (3.28)	15 (5.49)	17 (6.23)	
hadm_30	8.76 (4.15, 20.92)	6.36 (3.33, 17.73)	7.85 (3.65, 15.64)	7.79 (3.99, 18.81)	10.45 (4.96, 23.77)	12.63 (6.23, 25.93)	**<0.0001**
status_30							**<0.0001**
Alive	1,102 (80.73)	241 (88.28)	244 (89.38)	220 (80.59)	211 (77.29)	186 (68.13)	
Death	263 (19.27)	32 (11.72)	29 (10.62)	53 (19.41)	62 (22.71)	87 (31.87)	
hadm_90	8.76 (4.15, 20.92)	6.36 (3.33, 17.73)	7.85 (3.65, 15.64)	7.79 (3.99, 18.81)	10.45 (4.96, 23.77)	12.63 (6.23, 25.93)	**<0.0001**
status_90							**<0.0001**
Alive	1,047 (76.70)	232 (84.98)	231 (84.62)	209 (76.56)	200 (73.26)	175 (64.10)	
Death	318 (23.30)	41 (15.02)	42 (15.38)	64 (23.44)	73 (26.74)	98 (35.90)	
hadm_365	8.76 (4.15, 20.92)	6.36 (3.33, 17.73)	7.85 (3.65, 15.64)	7.79 (3.99, 18.81)	10.45 (4.96, 23.77)	12.63 (6.23, 25.93)	**<0.0001**
status_365							**<0.0001**
Alive	981 (71.87)	222 (81.32)	219 (80.22)	197 (72.16)	182 (66.67)	161 (58.97)	
Death	384 (28.13)	51 (18.68)	54 (19.78)	76 (27.84)	91 (33.33)	112 (41.03)	
los_icu_day	3.41 (1.75, 7.24)	2.70 (1.47, 5.46)	2.89 (1.60, 6.17)	3.30 (1.74, 7.22)	3.94 (1.86, 7.81)	4.93 (2.24, 9.49)	**<0.0001**
status_icu							**<0.01**
Alive	1,233 (90.33)	255 (93.41)	251 (91.94)	248 (90.84)	249 (91.21)	230 (84.25)	
Death	132 (9.67)	18 (6.59)	22 (8.06)	25 (9.16)	24 (8.79)	43 (15.75)	
los_hospital_day	8.01 (4.00, 16.14)	6.56 (3.23, 13.82)	6.73 (3.68, 12.85)	7.42 (3.77, 15.61)	8.93 (4.66, 17.76)	11.80 (6.01, 20.66)	**<0.0001**
status_hospital							**<0.0001**
Alive	1,181 (86.52)	247 (90.48)	251 (91.94)	241 (88.28)	233 (85.35)	209 (76.56)	
Death	184 (13.48)	26 (9.52)	22 (8.06)	32 (11.72)	40 (14.65)	64 (23.44)	

PIV was an independent risk factor for ACM in critically ill ischemic stroke patients during hospitalization, 30 days, 90 days and 365 days after ICU hospitalization. SBP, systolic blood pressure; DBP, diastolic blood pressure; MBP, mean arterial pressure; RP, respiratory rate; T, body temperature; SPO_2_, oxygen saturation; COPD, chronic obstructive pulmonary disease; HF, heart failure; PIV, pan-immune inflammation index; PLT, platelet count; NEU, neutrophil count; LYM, lymphocyte count; MONO, monocyte count; WBC, white blood cell; INR, international normalized ratio; PT, prothrombin time; ALT, alanine transaminase; AST, aspartate transaminase; ALP, alkaline phosphatase; GCS, Glasgow Coma Scale; CCI, Charlson comorbidity index; SOFA, Sequential Organ Failure Assessment; SAPSII, Simplified Acute Physiology Score; APSIII, Acute Physiology and Chronic Health II Score; OASIS, Oxford Acute Disease Severity Score; Braden, Braden Assessment Scale; surgery, interventional therapy; hadm_30, actual survival time within 30 days of admission for enrolled patients; hadm_90, actual survival time within 90 days of admission for enrolled patients; hadm_365, actual survival time within 365 days of admission for enrolled patients; status_30, survival status within 30 days of admission for enrolled patients; status_90, survival status within 90 days of admission for enrolled patients; status_365, survival status within 365 days of admission for enrolled patients; los_icu_day, ICU stay duration for enrolled patients; los_hospital_day, length of hospital stay for enrolled patients; status_icu, survival status during ICU hospital stay for enrolled patients; status_hospital, survival status during hospital stay for enrolled patients. Values with *p* < 0.05 are in bold.

**Figure 2:**
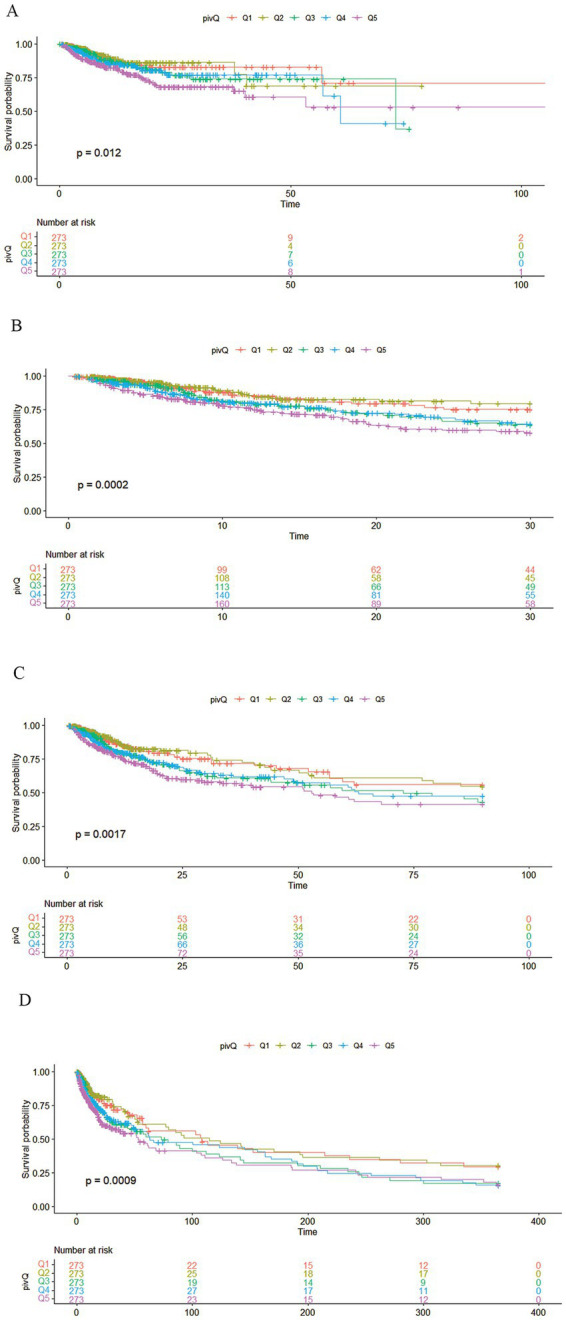
Kaplan–Meier survival analysis curve. **(A)** Kaplan–Meier survival analysis curve of critically ill ischemic stroke patients during hospitalization; pivQ: PIV grouped into quintiles Q1–Q5. **(B)** 30-day Kaplan–Meier survival analysis curve of critically ill ischemic stroke patients; pivQ: PIV grouped into quintiles Q1–Q5. **(C)** 90-day Kaplan–Meier survival analysis curve of critically ill ischemic stroke patients; pivQ: PIV grouped into quintiles Q1–Q5. **(D)** 365-day Kaplan–Meier survival analysis curve of critically ill ischemic stroke patients; pivQ: PIV grouped into quintiles Q1–Q5.

**Figure 3:**
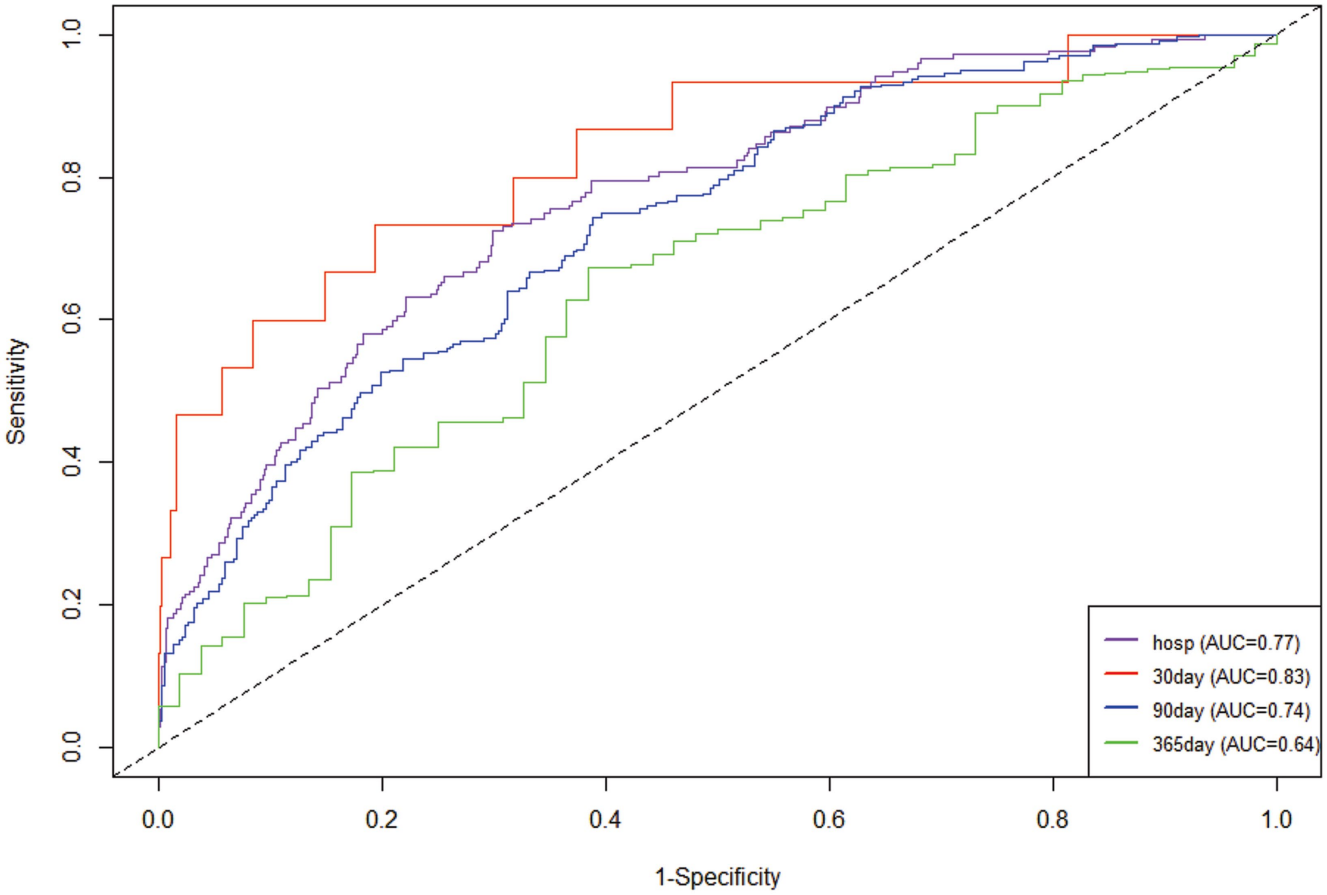
Time-dependent ROC curves were plotted according to the Cox model.

**Figure 4:**
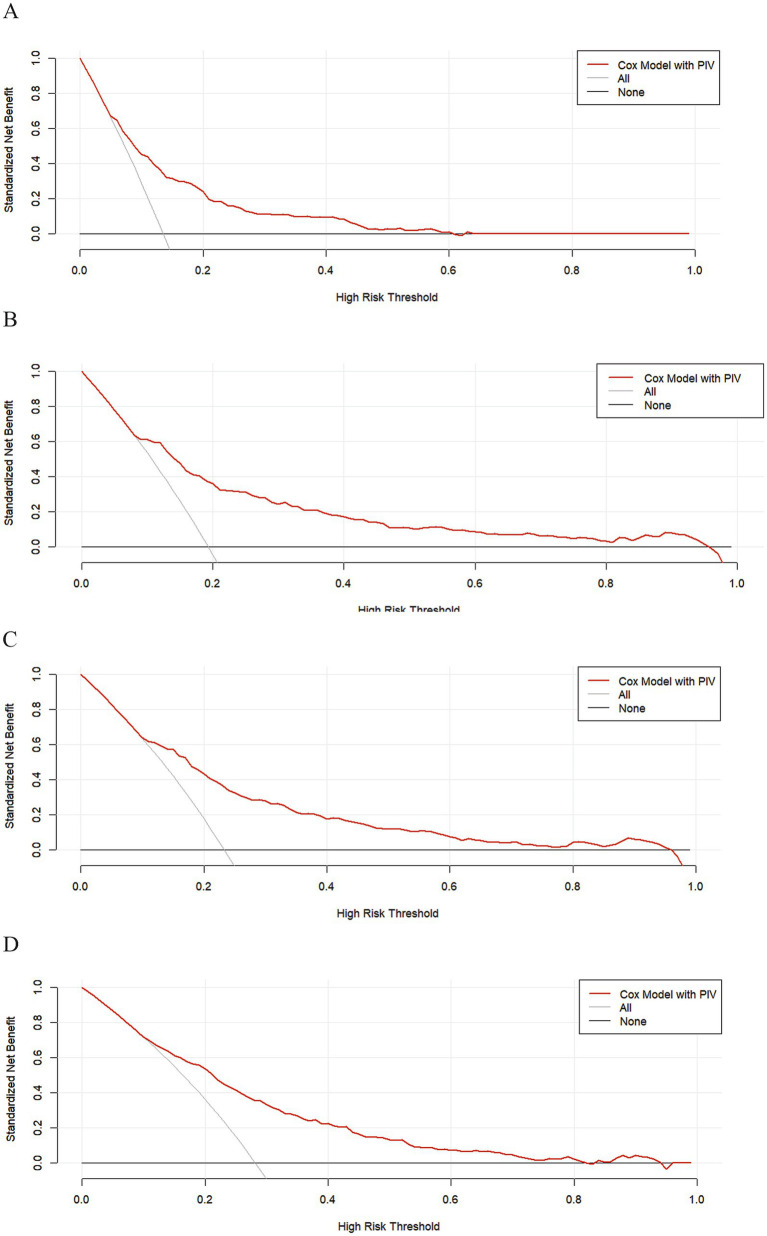
The DCA clinical decision curve, based on the COX model, was performed during hospitalization and at 30 days, 90 days, and 365 days post-hospitalization. **(A)** During hospitalization, **(B)** at 30 days, **(C)** at 90 days, and (D) at 365 days post-hospitalization.

The research variables to be included first underwent univariate Cox regression analysis, followed by the inclusion of covariates with *p* < 0.05 and potential risk factors in multivariate Cox regression analysis. Table 2 shows the adjusted analyses of PIV and intra-hospital, 30-day, 90-day, and 365-day post hospitalization ACM for critically ill patients with ischemic stroke using the Cox proportional hazards model. The results indicate that, regardless of whether adjustments were made, elevated PIV consistently correlates with in-hospital [risk ratio (HR): 1.98; 95% confidence interval CI: (1.19–3.29); *p* = 0.01], 30-day [risk ratio (HR): 2.56; 95% confidence interval CI: (1.65–3.97); *p* < 0.0001], 90-day [risk ratio (HR): 1.97; 95% confidence interval CI: (1.35, 2.89); *p* < 0.001] and 365-day [risk ratio (HR): 1.76; 95% confidence interval CI: (1.25–2.48); *p* = 0.001] ACM were significantly associated. The use of K–M survival analysis helped examine the relationship of different PIV quintiles with outcomes. As shown in Figure 2, as the survival curves showed higher mortality risk and lower survival during hospitalization, 30 days, 90 days, and 365 days after hospitalization. According to Figure 3, the final predictive model constructed in this study demonstrated good predictive efficacy for hospitalization, 30-day and 90-day post-hospitalization ACM in ischemic stroke patients. In addition, Figure 4 shows that the Cox model ultimately constructed in this study demonstrates high predictive value for predicting ACM risk during hospitalization, at 30 days, 90 days, and 365 days post hospitalization, especially under lower and moderate-high risk thresholds. This indicates that the model developed in this study has potential application value in clinical practice, helping doctors more accurately identify high-risk patients who require focused attention and intervention, thereby reducing the risk of patient death.

**Table 2:** Cox proportional risk ratio of critically ill stroke patients at in-hospital, 30 days, 90 days, and 365 days.

Categories hospital mortality	Crude model		Model 1		Model 2		Model 3	
Character	95% CI	*p*	95% CI	*p*	95% CI	*p*	95% CI	*p*
Q2	ref		ref		ref		ref	
Q1	1.11 (0.63, 1.96)	0.72	1.13 (0.64, 2.00)	0.68	1.12 (0.63, 1.99)	0.71	1.03 (0.57, 1.85)	0.92
Q3	1.33 (0.77, 2.29)	0.30	1.43 (0.83, 2.46)	0.20	1.44 (0.83, 2.51)	0.19	1.54 (0.88, 2.69)	0.13
Q4	1.49 (0.88, 2.51)	0.13	1.57 (0.93, 2.65)	0.09	1.5 (0.88, 2.56)	0.14	1.43 (0.83, 2.47)	0.20
Q5	2.05 (1.26, 3.34)	0.004	2.16 (1.33, 3.53)	0.002	2.1 (1.27, 3.47)	0.004	1.98 (1.19, 3.29)	0.01

Categories 30 days mortality	Crude model		Model 1		Model 2		Model 3	
Character	95% CI	*p*	95% CI	*p*	95% CI	*p*	95% CI	*p*
Q2	ref		ref		ref		ref	
Q1	1.15 (0.70, 1.91)	0.57	1.28 (0.78, 2.12)	0.33	1.31 (0.79, 2.17)	0.30	1.31 (0.78, 2.18)	0.30
Q3	1.74 (1.11, 2.74)	0.02	1.97 (1.25, 3.10)	0.003	2 (1.26, 3.17)	0.003	2.12 (1.34, 3.37)	0.001
Q4	1.81 (1.17, 2.82)	0.01	2 (1.28, 3.11)	0.002	1.95 (1.24, 3.05)	0.004	1.85 (1.17, 2.93)	0.01
Q5	2.31 (1.51, 3.51)	<0.0001	2.74 (1.79, 4.18)	<0.0001	2.65 (1.71, 4.09)	<0.0001	2.56 (1.65, 3.97)	<0.0001

Categories 90 days mortality	Crude model		Model 1		Model 2		Model 3	
Character	95% CI	*p*	95% CI	*p*	95% CI	*p*	95% CI	*p*
Q2	ref		ref		ref		ref	
Q1	1.04 (0.68, 1.60)	0.86	1.16 (0.75, 1.78)	0.51	1.19 (0.77, 1.84)	0.43	1.16 (0.75, 1.80)	0.51
Q3	1.49 (1.01, 2.20)	0.04	1.67 (1.13, 2.47)	0.01	1.69 (1.14, 2.51)	0.01	1.7 (1.14, 2.54)	0.01
Q4	1.49 (1.02, 2.18)	0.04	1.61 (1.10, 2.36)	0.01	1.57 (1.07, 2.31)	0.02	1.46 (0.98, 2.15)	0.06
Q5	1.88 (1.31, 2.69)	<0.001	2.23 (1.55, 3.20)	<0.0001	2.16 (1.48, 3.14)	<0.0001	1.97 (1.35, 2.89)	<0.001

Categories 365 days mortality	Crude model		Model 1		Model 2		Model 3	
Character	95% CI	*p*	95% CI	*p*	95% CI	*p*	95% CI	*p*
Q2	ref		ref		ref		ref	
Q1	1.05 (0.72, 1.54)	0.80	1.15 (0.78, 1.69)	0.47	1.2 (0.81, 1.76)	0.36	1.12 (0.76, 1.66)	0.56
Q3	1.49 (1.05, 2.11)	0.03	1.67 (1.18, 2.37)	0.004	1.63 (1.14, 2.33)	0.01	1.54 (1.08, 2.21)	0.02
Q4	1.52 (1.08, 2.12)	0.02	1.61 (1.15, 2.26)	0.01	1.54 (1.09, 2.17)	0.01	1.38 (0.98, 1.95)	0.07
Q5	1.81 (1.31, 2.51)	<0.001	2.15 (1.55, 2.98)	<0.0001	2.03 (1.45, 2.85)	<0.0001	1.76 (1.25, 2.48)	0.001

COPD, chronic obstructive pulmonary disease; HF, heart failure; PIV, pan-immune inflammatory value; INR, international normalized ratio; PT, prothrombin time; ALT, alanine transaminase; ALP, alkaline phosphatase; GCS, Glasgow Coma Scale; CCI, Charlson comorbidity index; surgery, endovascular interventional therapy; crude model, original model (unadjusted); Model 1: PIV, age, sex; Model 2: PIV, age, sex, hypertension, fibration, hyperlipemia, diabetes, COPD, HF, INR, PT, AST, ALT, ALP; Model 3: PIV, age, sex, hypertension, fibration, hyperlipemia, diabetes, COPD, HF, INR, PT, AST, ALT, ALP, GCS, CCI, antiplate, anticoagulation, surgery, thrombolysis.

The restricted cubic spline plot showed that PIV was associated with a “J-shaped curve” relationship with ACM in critically ischemic stroke patients during hospitalization, and 30 days, 90 days and 365 days after hospitalization.

**Figure 5:**
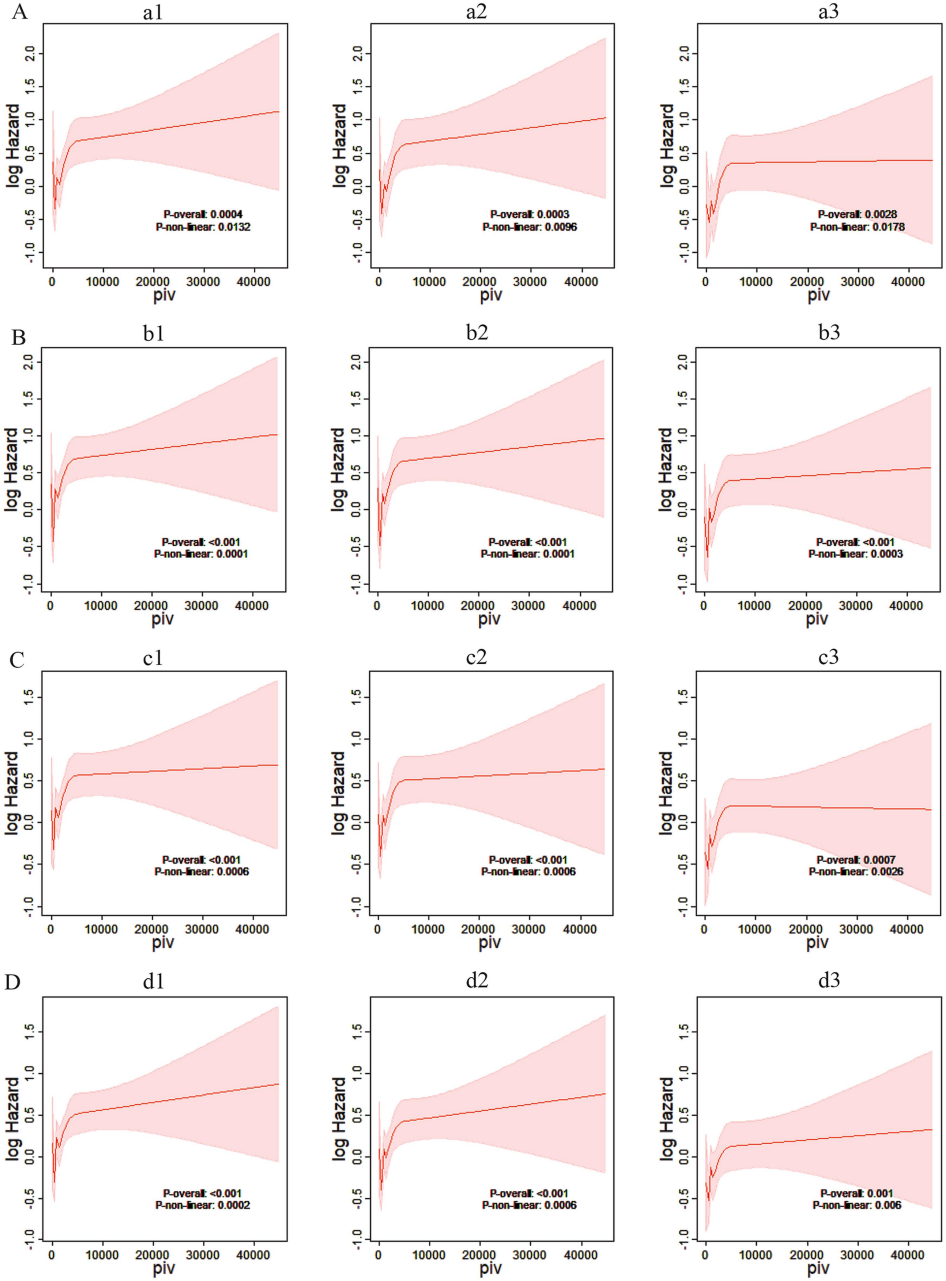
Restricted cubic spline plots. **(A)** Restricted cubic spline plots based on in-hospital Cox proportional risk ratios of critically ill stroke patients. **(a1)** Restricted cubic spline plots based on in-hospital Cox proportional risk ratios of Model 1. **(a2)** Restricted cubic spline plots based on in-hospital Cox proportional risk ratios of Model 2. **(a3)** Restricted cubic spline plots based on in-hospital Cox proportional risk ratios of Model 3; log hazard, log risk ratio; piv, pan-immune inflammatory index. **(B)** Based on the restricted cubic spline plot of the 30-day Cox proportion risk ratio for critically ill stroke patients. **(b1)** The restricted cubic spline plot of the 30-day Cox proportion risk ratio for Model 1. **(b2)** The restricted cubic spline plot of the 30-day Cox proportion risk ratio for Model 2. **(b3)** The restricted cubic spline plot of the 30-day Cox proportion risk ratio for Model 3; log hazard, log risk ratio; piv, panimmune inflammation index. **(C)** Based on the restricted cubic spline plot of the 90-day Cox proportion risk ratio for critically ill stroke patients. **(c1)** The restricted cubic spline plot of the 90-day Cox proportion risk ratio for Model 1. **(c2)** The restricted cubic spline plot of the 90-day Cox proportion risk ratio for Model 2. **(c3)** The restricted cubic spline plot of the 90-day Cox proportion risk ratio for Model 3; log hazard, log risk ratio; piv, pan-immune inflammation index. **(D)** Based on the restricted cubic spline plot of 365-day Cox risk ratio for critically ill stroke patients. **(d1)** The restricted cubic spline plot of 365-day Cox risk ratio for Model 1. **(d2)** The restricted cubic spline plot of 365-day Cox risk ratio for Model 2. **(d3)** The restricted cubic spline plot of 365-day Cox risk ratio for Model 3; log hazard, log risk ratio; piv, panimmune inflammation index.

According to Figure 5, the correlation between PIV and ACM in critically ill ischemic stroke patients at hospitalization, and 30 days, 90 days, and 365 days post hospitalization was investigated using restricted cubic spline plots. The results show that in either univariate Cox models or multivariate Cox models adjusted for corresponding variables, PIV exhibited a “J-shaped curve” relationship with ACM in critically ill ischemic stroke patients during hospitalization, and 30 days, 90 days, and 365 days post hospitalization. As PIV increases, the risk of death within these time points shows a non-linear increase (*p*-non-linear <0.05). To avoid the impact of age on PIV, this study included age as a continuous variable in Model 1 and adjusted for it to plot the RCS curve. Our RCS curve strongly demonstrates that the J-shaped risk pattern remains unchanged even after strict age adjustment, further supporting the stability of our findings (Supplementary Figure 1).

### Subgroup analysis

**Figure 6:**
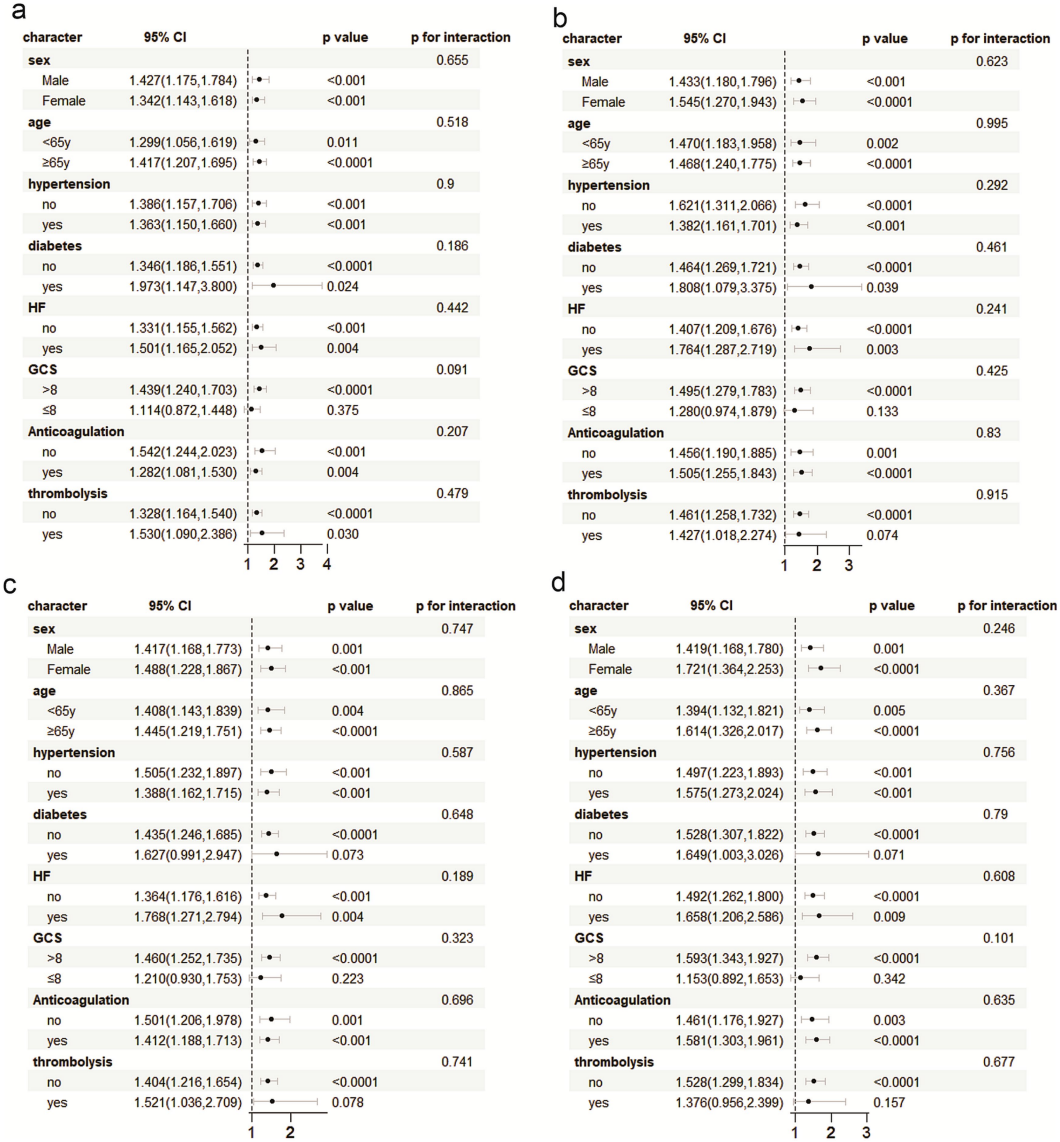
Subgroup analysis was performed with ACM during hospitalization, and 30 days, 90 days, and 365 days post hospitalization. **(a)** hospitalization, and **(b)** 30 days, **(c)** 90 days, and **(d)** 365 days post hospitalization.

The prognostic utility of PIV in predicting critical ischemic stroke patients was carefully evaluated in various patient subgroups, including gender, age and hypertension, diabetes, heart failure, anticoagulant use, intravenous thrombolysis, and the Glass Coma Scale (GCS). In order to further exclude the interference of age, this study divided the population into two levels according to age ≥65 years and <65 years, and conducted interaction analysis to further confirm that the J-type risk association persisted in the two subgroups (interaction *p*-value >0.05), indicating that age had no statistically significant effect on PIV results (Supplementary Figure 2). PIV was an important predictor of increased risk of death during hospitalization, and 30, 90 and 365 days after hospitalization. The analysis of interaction showed that PIV predictive ability was robust, and no significant interaction between groups was detected (*p* for interaction all >0.05) (as shown in Figure 6).

## Discussion

As our understanding of the pathophysiological mechanisms of stroke development deepens, the concept of the ischemic penumbra has brought about a milestone change in ischemic stroke treatment, significantly improving survival rates and outcomes for stroke patients (26). The ischemic penumbra refers to the brain tissue surrounding the infarcted core. This tissue has blood perfusion below the level required for normal function, but above the threshold for structural damage. The significance of it lies in its dynamic reversibility and its role as the primary target for acute stroke therapies. However, due to the sensitivity of brain tissue to ischemia and hypoxia and its irreversible nature, treatment measures are greatly constrained by the time window. Both intravenous thrombolysis and endovascular interventions require restoring blood flow to hypo perfused brain regions within a specified time window, necessitating early application after the onset of ischemic stroke. Limited treatment time and a series of contraindications make it difficult for current treatment technologies to cover most stroke populations. Therefore, expanding the treatment window and exploring new therapeutic approaches are topics of great interest.

The mechanisms of stroke development are diverse, with inflammation and immune responses being two major recognized factors that drive damage progression and lead to long-term poor outcomes. Inflammatory responses induce the release of reactive oxygen species (ROS) and promote immune-derived mechanisms associated with cytotoxicity and brain injury. After a stroke occurs, local damaged tissues initiate an inflammatory cascade reaction, leading to the proliferation of numerous inflammatory cells and the release of inflammatory mediators. This triggers abnormal activation of microglia and astrocytes, which in turn increases the permeability of the blood–brain barrier (BBB), allowing many inflammatory cells to infiltrate locally and release harmful substances. This can exacerbate brain edema and tissue necrosis, while also suppressing immune function, leading to a series of adverse outcomes such as post-stroke infections, dementia, formation of small vessel disease, hemorrhagic transformation, early neurological deterioration, recurrent strokes, and even death (27–38).

Focusing on the inflammatory immune response following stroke, researchers continuously ponder whether correcting the body’s inflammatory immune imbalance can protect brain tissue from further damage, aiming to improve patient outcomes. A series of neuroprotective agents have gradually entered clinical research stages. For example, the ESCAPE trial used peptides that interfere with postsynaptic density protein 95 (PSD95), providing evidence that neuroprotection is feasible in human stroke treatment (39).

Ischemic brain injury leads to the release of immunologically active molecules (damage-associated molecular patterns, DAMPs). These molecules locally activate inflammatory immune cells such as microglia and astrocytes. Additionally, DAMPs released into the bloodstream can promote the recruitment of circulating inflammatory immune cells to the brain and activate complex peripheral inflammatory immune responses in stroke (5). PIV is a composite index of immune inflammation involving monocytes, neutrophils, platelets and lymphocytes in peripheral blood. It also combines the neutrophil-to-lymphocyte ratio (NLR), monocyte-to-lymphocyte ratio (MLR), and platelet-to-lymphocyte ratio (PLR). Numerous studies have shown that PIV has good predictive value for diseases in multiple areas, including cardiovascular and cerebrovascular diseases, cancer, psoriasis, and rheumatoid arthritis (40–45).

In this study, we investigated the correlation between PIV and mortality risk in patients with acute ischemic stroke from the ICU. The results showed that elevated PIV is associated with increased ACM during hospitalization and at 30 days, 90 days, and 365 days post hospitalization. Even after adjusting for confounding risk factors, PIV still maintains a certain correlation with ACM in this population. Combining current research in relevant fields, the possible mechanisms explaining high PIV leading to poor prognosis of ischemic stroke may be any of the following.

First, after a stroke occurs, ischemia and hypoxia in the damaged brain tissue trigger an inflammatory cascade reaction, inducing infiltration of circulating immune-inflammatory cells (such as neutrophils and monocytes) and inflammatory mediators (such as TNF-α) into the local tissues. These cells interact with vascular endothelial cells at the injury site, promoting the release of free oxygen radicals and matrix metalloproteinase-9 (MMP-9), which subsequently leads to intracranial hemorrhage transformation, early neurological deterioration, and malignant brain edema, among other adverse reactions, resulting in poor patient outcomes (46–49). At the same time, under inflammatory stimulation, activated platelets interact directly with neutrophils and monocytes by altering the surface expression of P-selectin or CD40, leading to the formation of malignant thrombi and increasing the risk of vascular occlusion (13). The increase in neutrophils, monocytes, and platelets may result in elevated PIV levels; the more severe the inflammatory response, the higher the PIV value, thus indicating a positive correlation between high PIV and poor patient outcomes.

Second, numerous basic studies have shown that abnormal inflammatory responses after a stroke can easily lead to immune-inflammatory imbalance, causing significant loss of immune cells (lymphocytes) and immune factors, leading to immunosuppression and subsequent poor outcomes (5, 48, 50–52). Lymphocytes, as important immune cells in the body, when reduced, result in an increase in PIV levels, reflecting to some extent the dysfunction of the body’s immune system.

It is worth noting that age-related myeloid skewness represents a complex physiological phenomenon involving alterations in hematopoietic stem cell differentiation potential, modifications to the bone marrow microenvironment, and functional decline of the immune system. This mechanism may lead to increased neutrophils, monocytes, and platelets while decreasing lymphocytes. Such biological mechanisms could limit the predictive capacity of pulmonary interstitial pneumonia (PIV) in certain elderly populations. The study strongly confirmed that age did not significantly interfere with the correlation between PIV and clinical outcomes through interaction analysis by age stratification (Supplementary Figure 2) and drawing of RCS curves with age as a continuous variable into adjustment (Supplementary Figure 1).

Additionally, stroke treatment typically involves thrombolytic therapy and antiplatelet therapy, both of which affect platelet count and function. Thrombolytic therapy may induce thrombocytopenia, while aspirin or dual antiplatelet therapy might impair platelet reactivity. To eliminate the interference of platelets in the research findings, this study conducted sensitivity analysis to re-examine the relationship between PIV without platelet count (designated as pivmp) and clinical outcomes. The results confirmed that PIV’s predictive capacity remains robust and independent of platelet-specific changes (whether intrinsic or treatment-induced) (Supplementary Table 1).

This study found that elevated PIV is associated with high ACM in critically ill patients with ischemic stroke, consistent with previous research conclusions in this field. Compared to previous studies, this study extended the follow-up period to 1 year after hospitalization, exploring the long-term prognosis of PIV in ischemic stroke populations, filling a gap in related research. Additionally, using a multicenter database, the data sources and sample sizes are more comprehensive and representative compared to single-center studies, providing a broader and more accurate reflection of the relationship between PIV and the prognosis of ischemic stroke. The association between serological inflammatory immune markers and clinical outcomes in stroke patients has been increasingly confirmed, offering new insights for the development of relevant clinical drugs. As the mechanisms become better understood, neuroprotective agents aimed at suppressing systemic inflammation and improving immune suppression continue to be developed, achieving positive feedback in various animal experiments, bringing new hope for stroke treatment options (53–55).

However, this study utilized a retrospective analysis, and despite multivariate adjustments and subgroup analyses, residual confounding remains possible. Additionally, the Glasgow Coma Scale (GCS) though standard for assessment of ischemic stroke patients, retains subjectivity which can lead to selection bias. Third, other serum indicators such as glucose and albumin are important confounders but were not included in this evaluation due to missing values exceeding 20%. Finally, this study only explored the correlation between baseline PIV and ACM in ischemic stroke patients, without investigating its dynamic fluctuations throughout the disease process and their relationship with prognosis. The limitations stated require further exploration and improvement by subsequent studies.

## Conclusion

This study demonstrates that PIV can reflect the inflammatory response state of critically ill patients with ischemic stroke to some extent, and may be an independent predictor of ACM in such patients. Incorporating PIV into clinical practice could provide additional support for diagnosing and managing ischemic stroke patients in the ICU, thereby improving the identification of high-risk patients and promoting timely intervention.

## Data Availability

The raw data supporting the conclusions of this article will be made available by the authors, without undue reservation.

## Supplementary material

SUPPLEMENTARY FIGURE 1 The RCS curve is plotted according to the four prediction models stratified by age. **(a)** Age stratified RCS curve at 30 days post-discharge. **(b)** Age stratified RCS curve at 90 days post-discharge.**(c)** Age stratified RCS curve at 365 days post-discharge. **(d)** Age stratified RCS curve during hospitalization.

SUPPLEMENTARY FIGURE 2 Subgroup analysis and interaction analysis of the prediction model were carried out according to age stratification. **(a)** Age stratified subgroup analysis and interactions at 30 days post-discharge. **(b)** Age stratified subgroup analysis and interactions at 90 days post-discharge. **(c)** Age stratified subgroup analysis and interactions at 365 days post-discharge. **(d)** Age stratified subgroup analysis and interactions during hospitalization.
