# Supplementary figures and images for: Association between pan-immune inflammatory value and all-cause mortality in critically ill patients with ischemic stroke: a retrospective analysis of the MIMIC-IV database (2008–2022)
Source: Front Neurol. 2025 Aug 18;16:1644817. doi: 10.3389/fneur.2025.1644817 (PMC12400861; doi:10.3389/fneur.2025.1644817)

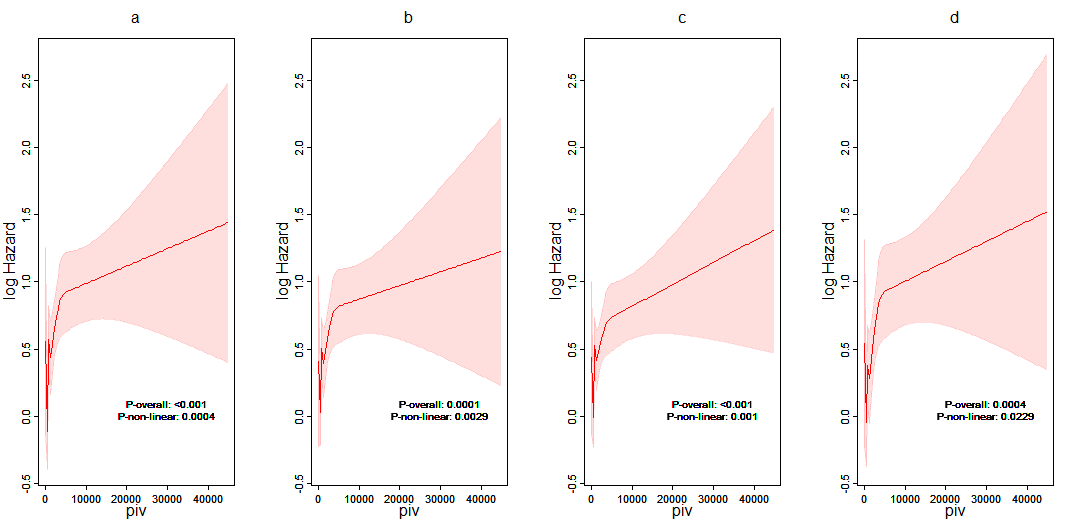

Supplement: SUPPLEMENTARY FIGURE 1 — The RCS curve is plotted according to the four prediction models stratified by age. (a) Age stratified RCS curve at 30 days post-discharge. (b) Age stratified RCS curve at 90 days post-discharge.(c) Age stratified RCS curve at 365 days post-discharge. (d) Age stratified RCS curve during hospitalization. [file Image_1.tiff]

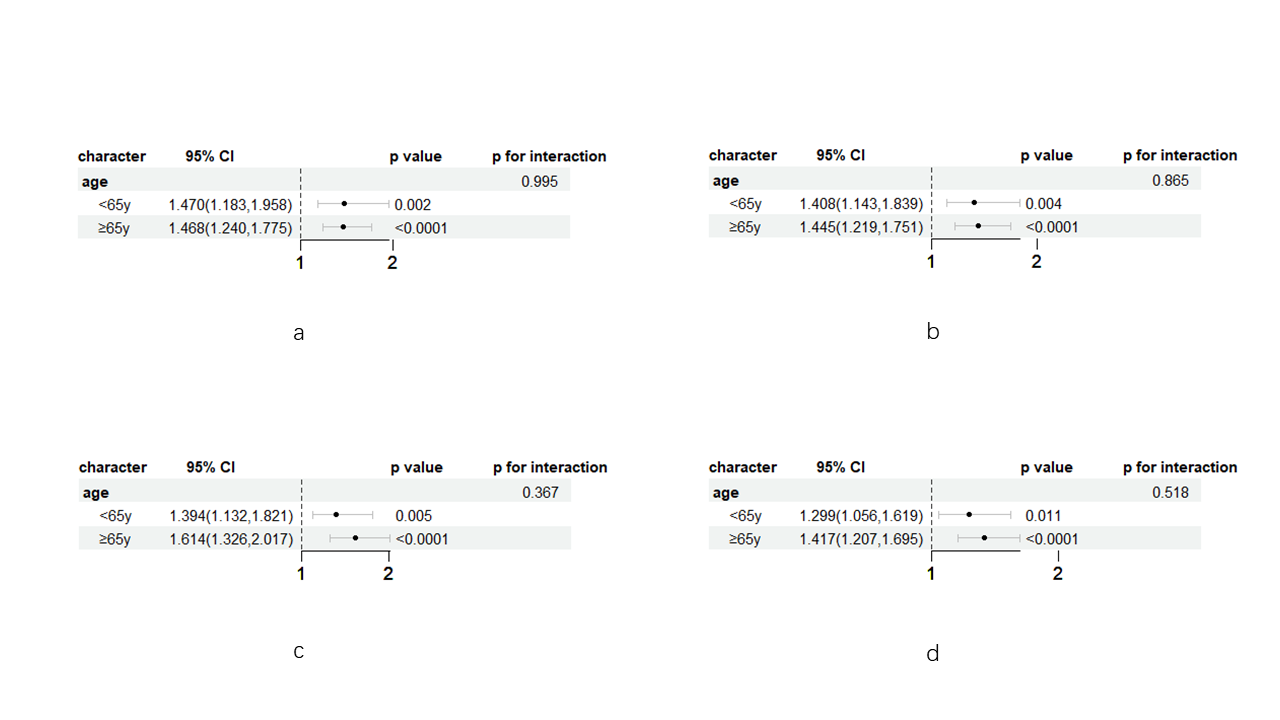

Supplement: SUPPLEMENTARY FIGURE 2 — Subgroup analysis and interaction analysis of the prediction model were carried out according to age stratification. (a) Age stratified subgroup analysis and interactions at 30 days post-discharge. (b) Age stratified subgroup analysis and interactions at 90 days post-discharge. (c) Age stratified subgroup analysis and interactions at 365 days post-discharge. (d) Age stratified subgroup analysis and interactions during hospitalization. [file Image_2.tif]
