# Supplementary material for: Association between pan-immune inflammatory value and all-cause mortality in critically ill patients with ischemic stroke: a retrospective analysis of the MIMIC-IV database (2008–2022)
Source: Front Neurol. 2025 Aug 18;16:1644817. doi: 10.3389/fneur.2025.1644817 (PMC12400861; doi:10.3389/fneur.2025.1644817)
Supplement: Supplementary file 3 [file Table_1.docx]

Supplemental table 1

|  | **pivmpQ** | | | | | | | |
| --- | --- | --- | --- | --- | --- | --- | --- | --- |
| **categories  hospital mortality** | crude model | | Model 1 | | Model 2 | | Model 3 | |
| character | 95%CI | P | 95%CI | P | 95%CI | P | 95%CI | P |
| Q2 | ref |  | ref |  | ref |  | ref |  |
| Q1 | 1.29(0.69,2.41) | 0.43 | 1.31(0.70,2.45) | 0.40 | 1.39(0.74,2.62) | 0.31 | 1.17(0.62,2.21) | 0.63 |
| Q3 | 1.69(0.95,3.01) | 0.08 | 1.78(1.00,3.17) | 0.05 | 1.8(1.00,3.24) | 0.05 | 1.77(0.98,3.18) | 0.06 |
| Q4 | 1.77(1.01,3.09) | 0.05 | 1.83(1.05,3.20) | 0.03 | 1.91(1.08,3.37) | 0.03 | 1.82(1.03,3.21) | 0.04 |
| Q5 | 2.94(1.75,4.94) | <0.0001 | 3.09(1.84,5.19) | <0.0001 | 3.24(1.90,5.53) | <0.0001 | 2.78(1.63,4.76) | <0.001 |
| p for trend |  | <0.0001 |  | <0.0001 |  | <0.0001 |  | <0.0001 |

|  | **pivmpQ** | | | | | | | |
| --- | --- | --- | --- | --- | --- | --- | --- | --- |
| **categories  30 days mortality** | crude model | | Model 1 | | Model 2 | | Model 3 | |
| character | 95%CI | P | 95%CI | P | 95%CI | P | 95%CI | P |
| Q2 | ref |  | ref |  | ref |  | ref |  |
| Q1 | 1.17(0.68,2.02) | 0.57 | 1.27(0.74,2.19) | 0.39 | 1.35(0.78,2.34) | 0.29 | 1.26(0.72,2.19) | 0.42 |
| Q3 | 2.1(1.31,3.35) | 0.002 | 2.25(1.41,3.59) | <0.001 | 2.3(1.43,3.69) | <0.001 | 2.23(1.39,3.60) | 0.001 |
| Q4 | 2.12(1.34,3.35) | 0.001 | 2.31(1.46,3.65) | <0.001 | 2.38(1.49,3.80) | <0.001 | 2.34(1.47,3.75) | <0.001 |
| Q5 | 2.79(1.81,4.31) | <0.0001 | 3.15(2.04,4.87) | <0.0001 | 3.29(2.10,5.15) | <0.0001 | 2.87(1.83,4.50) | <0.0001 |
| p for trend |  | <0.0001 |  | <0.0001 |  | <0.0001 |  | <0.0001 |

|  | **pivmpQ** | | | | | | | |
| --- | --- | --- | --- | --- | --- | --- | --- | --- |
| **categories  90 days mortality** | crude model | | Model 1 | | Model 2 | | Model 3 | |
| character | 95%CI | P | 95%CI | P | 95%CI | P | 95%CI | P |
| Q2 | ref |  | ref |  | ref |  | ref |  |
| Q1 | 1(0.63,1.60) | 1.00 | 1.02(0.64,1.63) | 0.94 | 1.05(0.65,1.70) | 0.83 | 0.93(0.57,1.49) | 0.75 |
| Q3 | 1.66(1.11,2.49) | 0.01 | 1.76(1.17,2.64) | 0.01 | 1.76(1.16,2.65) | 0.01 | 1.63(1.08,2.46) | 0.02 |
| Q4 | 1.81(1.23,2.67) | 0.003 | 1.91(1.30,2.82) | 0.001 | 1.95(1.32,2.89) | <0.001 | 1.84(1.24,2.74) | 0.003 |
| Q5 | 2.27(1.57,3.29) | <0.0001 | 2.44(1.69,3.54) | <0.0001 | 2.5(1.71,3.65) | <0.0001 | 2.1(1.43,3.09) | <0.001 |
| p for trend |  | <0.0001 |  | <0.0001 |  | <0.0001 |  | <0.0001 |

|  | **pivmpQ** | | | | | | | |
| --- | --- | --- | --- | --- | --- | --- | --- | --- |
| **categories  365 days mortality** | crude model | | Model 1 | | Model 2 | | Model 3 | |
| character | 95%CI | P | 95%CI | P | 95%CI | P | 95%CI | P |
| Q2 | ref |  | ref |  | ref |  | ref |  |
| Q1 | 0.96(0.63,1.45) | 0.84 | 0.96(0.63,1.45) | 0.83 | 1.01(0.66,1.53) | 0.98 | 0.87(0.57,1.34) | 0.53 |
| Q3 | 1.66(1.16,2.37) | 0.01 | 1.74(1.22,2.49) | 0.002 | 1.67(1.16,2.40) | 0.01 | 1.52(1.05,2.19) | 0.02 |
| Q4 | 1.74(1.23,2.45) | 0.002 | 1.82(1.29,2.57) | <0.001 | 1.84(1.30,2.61) | <0.001 | 1.69(1.19,2.40) | 0.004 |
| Q5 | 2.07(1.49,2.88) | <0.0001 | 2.21(1.59,3.07) | <0.0001 | 2.21(1.58,3.09) | <0.0001 | 1.82(1.29,2.56) | <0.001 |
| p for trend |  | <0.0001 |  | <0.0001 |  | <0.0001 |  | <0.0001 |

pivmpQ,Excluding the platelet count, it was calculated by the formula: (neutrophils x monocytes) / lymphocyte; crudel model: pivmpQ；model 1: pivmpQ, age, sex; model 2: pivmpQ, age, sex, hypertension, fibration, hyperlipemia, diabetes, COPD, HF, INR, PT, ALT, ALP; model 3: pivmpQ, age, sex, hypertension, fibration, hyperlipemia, diabetes, COPD, HF, INR, PT, ALT, ALP, GCS, CCI, Antiplate, Anticoagulation, surgery, thrombolysis;

Supplemental table 2

|  | **pivQ** | | | | | | | |
| --- | --- | --- | --- | --- | --- | --- | --- | --- |
| **categories hospital mortality** | crude model | | Model 1 | | Model 2 | | Model 3 | |
|  | 95%CI | P | 95%CI | P | 95%CI | P | 95%CI | P |
| Q2 | ref |  | ref |  | ref |  | ref |  |
| Q1 | 1.28(0.68,2.38) | 0.44 | 1.38(0.74,2.59) | 0.31 | 1.51(0.80,2.87) | 0.20 | 1.19(0.62, 2.28) | 0.59 |
| Q3 | 1.68(0.94,2.99) | 0.08 | 1.78(1.00,3.17) | 0.05 | 1.81(1.00,3.28) | 0.05 | 1.64(0.91, 2.94) | 0.10 |
| Q4 | 1.75(1.00,3.06) | 0.05 | 1.86(1.07,3.26) | 0.03 | 1.95(1.10,3.47) | 0.02 | 1.47(0.83, 2.61) | 0.19 |
| Q5 | 2.94(1.75,4.94) | <0.0001 | 3.14(1.87,5.28) | <0.0001 | 3.2(1.86,5.51) | <0.0001 | 1.96(1.13, 3.37) | 0.02 |
| p for trend |  | <0.0001 |  | <0.0001 |  | <0.0001 |  | 0.01 |

|  | **pivQ** | | | | | | | |
| --- | --- | --- | --- | --- | --- | --- | --- | --- |
| **categories 30 days mortality** | crude model | | Model 1 | | Model 2 | | Model 3 | |
|  | 95%CI | P | 95%CI | P | 95%CI | P | 95%CI | P |
| Q2 | ref |  | ref |  | ref |  | ref |  |
| Q1 | 1.15(0.70,1.91) | 0.57 | 1.28(0.78,2.12) | 0.33 | 1.33(0.80,2.22) | 0.28 | 1.3(0.77, 2.17) | 0.32 |
| Q3 | 1.74(1.11,2.74) | 0.02 | 1.97(1.25,3.10) | 0.003 | 2.06(1.29,3.27) | 0.002 | 1.93(1.21, 3.07) | 0.01 |
| Q4 | 1.81(1.17,2.82) | 0.01 | 2(1.28,3.11) | 0.002 | 1.99(1.27,3.14) | 0.003 | 1.75(1.10, 2.78) | 0.02 |
| Q5 | 2.31(1.51,3.51) | <0.0001 | 2.74(1.79,4.18) | <0.0001 | 2.7(1.74,4.19) | <0.0001 | 1.93(1.23, 3.01) | 0.004 |
| p for trend |  | <0.0001 |  | <0.0001 |  | <0.0001 |  | 0.003 |

|  | **pivQ** | | | | | | | |
| --- | --- | --- | --- | --- | --- | --- | --- | --- |
| **categories 90 days mortality** | crude model | | Model 1 | | Model 2 | | Model 3 | |
|  | 95%CI | P | 95%CI | P | 95%CI | P | 95%CI | P |
| Q2 | ref |  | ref |  | ref |  | ref |  |
| Q1 | 1.04(0.68,1.60) | 0.86 | 1.16(0.75,1.78) | 0.51 | 1.2(0.78,1.86) | 0.41 | 1.14(0.73,1.77) | 0.56 |
| Q3 | 1.49(1.01,2.20) | 0.04 | 1.67(1.13,2.47) | 0.01 | 1.72(1.15,2.55) | 0.01 | 1.55(1.04,2.32) | 0.03 |
| Q4 | 1.49(1.02,2.18) | 0.04 | 1.61(1.10,2.36) | 0.01 | 1.59(1.08,2.34) | 0.02 | 1.37(0.92,2.03) | 0.12 |
| Q5 | 1.88(1.31,2.69) | <0.001 | 2.23(1.55,3.20) | <0.0001 | 2.17(1.49,3.17) | <0.0001 | 1.55(1.05,2.28) | 0.03 |
| p for trend |  | <0.0001 |  | <0.0001 |  | <0.0001 |  | 0.02 |

|  | **pivQ** | | | | | | | |
| --- | --- | --- | --- | --- | --- | --- | --- | --- |
| **categories 365 days mortality** | crude model | | Model 1 | | Model 2 | | Model 3 | |
|  | 95%CI | P | 95%CI | P | 95%CI | P | 95%CI | P |
| Q2 | ref |  | ref |  | ref |  | ref |  |
| Q1 | 1.05(0.72,1.54) | 0.80 | 1.15(0.78,1.69) | 0.47 | 1.2(0.81,1.77) | 0.36 | 1.15(0.78,1.70) | 0.48 |
| Q3 | 1.49(1.05,2.11) | 0.03 | 1.67(1.18,2.37) | 0.004 | 1.65(1.15,2.35) | 0.01 | 1.45(1.01,2.09) | 0.04 |
| Q4 | 1.52(1.08,2.12) | 0.02 | 1.61(1.15,2.26) | 0.01 | 1.55(1.10,2.19) | 0.01 | 1.33(0.94,1.89) | 0.11 |
| Q5 | 1.81(1.31,2.51) | <0.001 | 2.15(1.55,2.98) | <0.0001 | 2.04(1.45,2.87) | <0.0001 | 1.45(1.02,2.06) | 0.04 |
| p for trend |  | <0.0001 |  | <0.0001 |  | <0.0001 |  | 0.03 |

pivQ；crudel model: pivQ; model 1: pivQ, age, sex; model 2: pivQ, age, sex, hypertension,fibration, hyperlipemia, diabetes, COPD, HF, INR, PT, ALT, AST ,ALP; model 3: pivQ, age, sex, hypertension, fibration, hyperlipemia, diabetes, COPD, HF, INR, PT, ALT, AST, ALP, GCS, CCI, Antiplate, Anticoag, surgery, thrombolysis, SOFA, OASIS, SAPSII, APSIII, Braden;

Supplemental table 3

| **variable** | **Total (n=1365)** | **Q1 (n=273)** | **Q2 (n=272)** | **Q3 (n=274)** | **Q4 (n=273)** | **Q5 (n=273)** |
| --- | --- | --- | --- | --- | --- | --- |
| **sex** |  |  |  |  |  |  |
| Female | 676(49.52) | 141(51.65) | 138(50.74) | 135(49.27) | 143(52.38) | 119(43.59) |
| Male | 689(50.48) | 132(48.35) | 134(49.26) | 139(50.73) | 130(47.62) | 154(56.41) |
| **age** |  |  |  |  |  |  |
| <65y | 560(41.03) | 110(40.29) | 97(35.66) | 126(45.99) | 116(42.49) | 111(40.66) |
| ≥65y | 805(58.97) | 163(59.71) | 175(64.34) | 148(54.01) | 157(57.51) | 162(59.34) |
| **hypertension** |  |  |  |  |  |  |
| no | 620(45.42) | 135(49.45) | 112(41.18) | 117(42.70) | 121(44.32) | 135(49.45) |
| yes | 745(54.58) | 138(50.55) | 160(58.82) | 157(57.30) | 152(55.68) | 138(50.55) |
| **diabetes** |  |  |  |  |  |  |
| no | 1175(86.08) | 232(84.98) | 221(81.25) | 240(87.59) | 238(87.18) | 244(89.38) |
| yes | 190(13.92) | 41(15.02) | 51(18.75) | 34(12.41) | 35(12.82) | 29(10.62) |
| **HF** |  |  |  |  |  |  |
| no | 1153(84.47) | 238(87.18) | 239(87.87) | 226(82.48) | 232(84.98) | 218(79.85) |
| yes | 212(15.53) | 35(12.82) | 33(12.13) | 48(17.52) | 41(15.02) | 55(20.15) |
| **GCS** |  |  |  |  |  |  |
| >8 | 115( 8.42) | 20( 7.33) | 16( 5.86) | 24( 8.79) | 25( 9.16) | 30(10.99) |
| ≤8 | 1250(91.58) | 253(92.67) | 257(94.14) | 249(91.21) | 248(90.84) | 243(89.01) |
| **Anticoagulation** |  |  |  |  |  |  |
| no | 468(34.29) | 87(31.87) | 76(27.94) | 101(36.86) | 105(38.46) | 99(36.26) |
| yes | 897(65.71) | 186(68.13) | 196(72.06) | 173(63.14) | 168(61.54) | 174(63.74) |
| **thrombolysis** |  |  |  |  |  |  |
| no | 1287(94.29) | 258(94.51) | 250(91.91) | 265(96.72) | 258(94.51) | 256(93.77) |
| yes | 78( 5.71) | 15( 5.49) | 22( 8.09) | 9( 3.28) | 15( 5.49) | 17( 6.23) |

(PIV: Q1:3.224-302.372; Q2:302.372-605.696; Q3:605.696-1052.635; Q4:1052.635-2017.179; Q5:2017.179-44699.253)
